# Impact of the Severe Malaria “Champions Program” on the Management of Severe Malaria Cases in 12 Hospitals of the North and Far North Regions of Cameroon

**DOI:** 10.4269/ajtmh.23-0528

**Published:** 2024-02-06

**Authors:** Eric Tchinda Meli, Yves-Marie Bernard, Joël Marcellin Ateba, Landry Tchoutang, Abas Mouliom, Annie Coriolan Ciceron, Jadmin Mostel, Christophe Tchadjeu, Olivier Palata, Mohamadu Wirngo, Aissata Fofana, Lawrence M. Barat

**Affiliations:** ^1^ U.S. President’s Malaria Initiative Impact Malaria Project, Jhpiego, Yaoundé, Cameroon;; ^2^ U.S. President’s Malaria Initiative Impact Malaria Project, Population Services International, Washington, District of Columbia;; ^3^ Programme National de Lutte contre le Paludisme, Yaoundé, Cameroon;; ^4^ U.S. President’s Malaria Initiative Impact Malaria Project, Association Camerounaise pour le Marketing Social, Yaoundé, Cameroon;; ^5^ U.S. President’s Malaria Initiative, U.S. Agency for International Development, Yaoundé, Cameroon

## Abstract

Malaria remains a main cause of morbidity and mortality in Cameroon. Since 2021, the U.S. President’s Malaria Initiative Impact Malaria Project has supported the National Malaria Control Program to develop the Champions program in two northern regions. We assessed this program’s preliminary effectiveness on the performance of hospitals in the management of severe malaria and reduction of malaria-related deaths. We conducted a secondary analysis of Outreach Training and Supportive Supervision (OTSS) data from four rounds (one round pre-Champions program and three rounds post-Champions program and 2020–2022 malaria-related mortality data for 12 hospitals). Using linear regressions, we measured changes in hospital readiness and competency of health workers in the management of severe malaria between baseline and subsequent rounds. There were statistically significant improvements in overall management of severe malaria scores in post-Champions OTSS rounds, with post-Champions round 3 exhibiting an increase of +14% (*P* = 0.013) over baseline. Overall health facility readiness scores exhibited an increase of +7% (*P* = 0.006) from baseline to post-Champions round 3. There were no statistically significant findings associated with providing the right treatment, as nearly all patients hospitalized with severe malaria were treated with a recommended severe malaria treatment. Reported inpatient malaria deaths and case fatality rates trended downward from 2020 to 2022, but these differences were not statistically significant. The Champions program resulted in significant improvements in quality of inpatient care for severe malaria. The downward trends in malaria deaths and case fatality rate will require further monitoring to determine whether the Champions program is having the desired impact of reducing inpatient deaths from malaria.

## INTRODUCTION

Despite declines in cases and deaths since 2000, malaria remains one of the main causes of morbidity and mortality in Cameroon. The percentage of hospital admissions that were diagnosed with malaria dropped in the general population from 41% in 2000 to 24.0% in 2014 and 23.6% in 2016 but then increased to 28% in 2019.^1^^–^^3^ In 2020, severe malaria cases accounted for 49% of all malaria cases reported in Cameroon (2020 National Malaria Control Program [NMCP] report, Cameroon Ministry of Health, unpublished), which is far higher than what is reported by the WHO for similar settings (between 3% and 10%). In the same year, the proportion of severe malaria cases reported by the NMCP in the North and the Far North were 56% and 47%, respectively. According to this report, the percentages of deaths attributed to malaria in children under 5 years were 44.8% and 48%, respectively, in the North and Far North regions, compared with 35.5% at the national level.

Since 2019, the U.S. President’s Malaria Initiative (PMI) Impact Malaria project has supported quality improvement for malaria case management in multiple countries, including implementation of Outreach Training and Supportive Supervision (OTSS), by using structured checklists to monitor the readiness of health facilities and competency of health workers in the management of uncomplicated malaria and the performance of malaria microscopy testing and rapid diagnostic tests (RDTs). The OTSS has been expanded under PMI Impact Malaria to include additional checklists for malaria in pregnancy and severe malaria.^4^ The Cameroon NMCP, with support from PMI Impact Malaria, launched and implemented OTSS to reinforce the quality of facility-based care, including inpatient management of severe malaria in the North and Far North regions, which carry the heaviest burden of malaria in the country. During OTSS visits, the readiness of the facilities as well as the competency of health workers is assessed through chart reviews of severe malaria inpatient cases (see Materials and Methods for more details). The data gathered through initial OTSS rounds identified a very small percentage of inpatient facilities that had health workers that were competent in the overall management of severe malaria (2% in round 1 and 3% in round 2), with poor scores in both initial assessment of patients and management of complications.^5^

Several studies conducted in similar settings have highlighted the gaps in inpatient care for severe malaria.^6^^–^^10^ A cross-sectional survey of health facilities in 11 districts in Uganda demonstrated that none of the inpatient facilities had all seven components of a basic care package for the management of severe malaria during the 3 months prior to the survey.^6^ Prompt care was reported for only 29% of patients, whereas severe malaria was correctly diagnosed in 27%. Elnour et al. reported that half of the health care providers (46.7%) in 20 hospitals in Gezira State, Sudan, did not receive training in severe malaria management, with just over half (55.4%) achieving a passing score on knowledge of severe malaria management.^7^ Overall compliance with severe malaria guidelines was just 2.2%.^7^

The MalariaCare project that preceded the PMI Impact Malaria project took important steps to address this issue of poor management of severe malaria by elaborating and implementing a series of tools—for mentoring on severe malaria (including triage, diagnosis, and treatment) and for assessing management of severe malaria—that were important for quality improvement.^11^^,^^12^ Based on the poor results documented during OTSS visits and building on the lessons learned from MalariaCare, PMI Impact Malaria worked with the Cameroon NMCP to develop and implement a more tailored quality improvement approach, as a supplement to OTSS, to address the poor performance of hospitals and their providers in severe malaria management. It did this by focusing on internal quality improvement activities to reinforce and sustain the quality of care for severe malaria cases with the goal of reducing malaria-related inpatient deaths. Since 2021, PMI Impact Malaria has worked with the NMCP and stakeholders to develop and implement this new approach, called the Champions program, in the hospitals in the Far North and North regions. The program included training selected clinicians from district hospitals on the management of severe malaria and its complications, equipping the clinicians with the skills to become Champions mentors in the management of severe malaria for other staff in their hospitals, and implementing internal quality assurance activities in their hospitals to improve the management of severe malaria. These activities included creating an internal quality committee to identify challenges in severe case management and solutions to address them and conducting monthly case reviews and death audits.

The U.S. President’s Malaria Initiative Impact Malaria coordinated with the NMCP and health districts, using health management information system (HMIS) data, to select the hospitals that reported the highest number of deaths from malaria to participate in this program. These activities targeted 11 district hospitals and five regional hospitals that serve as referral facilities for the whole population of the North (3,098,009 inhabitants) and the Far North (5,104,209 inhabitants) regions. While implementing the Champions program, the NMCP continued to implement regular OTSS visits as an external quality improvement approach using trained health district supervisors to monitor the quality of care for case management of severe malaria, as well as to monitor HMIS data on malaria-related deaths in the targeted hospitals.

### Champions program implementation process.

#### Training.

Thirty clinicians who were providing care to severe malaria cases in outpatient, internal medicine, and pediatric wards were prioritized for this training; 14 hospitals sent two participants each, while two hospitals sent one each. These clinicians were selected to be Champions candidates based on their willingness and interest in sharing the knowledge they acquired during training to mentor their colleagues and support improvements in the management of severe malaria in their respective hospitals. Training was conducted at the University Teaching Hospital (UTH) in Yaoundé to provide the best opportunity to improve the knowledge and skills of these clinicians on the identification and management of each complication through hands-on training supported by expert mentors from UTH. This training was conducted by eight mentors from the teaching hospital who were considered the best teachers, knowledgeable of the subject matter, and willing to contribute to the program. The final list of mentors was determined by the UTH leadership and the NMCP.

The training curriculum was developed based on WHO and national treatment guidelines, as well as the UTH curriculum.^13^ The training focused on the following: national and global malaria case management and prevention policies and guidelines, malaria epidemiology, physiology and pathology of malaria in vulnerable groups, and the management of severe febrile illness, including malaria and other conditions. The training also focused on critical care management of the complications of severe malaria, such as hypoglycemia, alteration of consciousness, seizures, and acid-base and fluid imbalances. Training on preparation and dosing of intravenous artesunate- and artemisinin-based combination therapy (ACT) follow-up treatment was also covered. The training lasted 2 weeks, which allowed enough time for both theory sessions and a practicum. For the practicum, participants took part in rotations on the internal medicine, pediatrics, and maternity services, participated in rounds with attending physicians, and discussed cases with mentors.

#### Posttraining activities.

After their training, all 30 trained clinicians became Champions who worked with their hospital and local health staff to create a quality committee, composed of the hospital staff, that coordinated a series of internal quality improvement activities aimed at reinforcing the capacity to manage cases of severe malaria. These activities encompassed the following:

1. A 2-day cascade training on severe malaria facilitated by the Champions for the hospital staff. Overall, 407 health workers participated in this training (hospital reports).
2. Monthly quality meetings during which charts of inpatient severe malaria cases were reviewed to identify and discuss gaps in care and to develop recommendations for the hospital management team. In the hospitals targeted, 119 charts were reviewed during the assessment period (hospital reports).
3. Monthly death audits to validate the appropriateness of the diagnosis of severe malaria, to correct the reporting of deaths, and to review the inpatient management and proposed solutions to gaps in care. A total of 42 deaths had been audited during the intervention period in the hospitals targeted (hospital reports).
4. Quarterly meeting of quality committees with district and regional health authorities to review quality and outcome data and to discuss possible corrective actions, such as the procurement of missing commodities and equipment and improvement in documentation.

The overall aim of this study was to evaluate the effect of the Champions program on the performance of hospitals in the management of severe malaria. More specifically, this study assessed trends in: 1) the readiness of targeted hospitals in the availability of critical tools before and after the implementation of the program; 2) the competency of health workers in these hospitals in the overall management of severe malaria before and after the launch of the program; 3) key subcomponents in the management of severe malaria (patient assessment, providing the right antimalarial treatment according to norms, and correctly treating the different complications from severe malaria); and 4) the number of deaths and the case fatality rate among patients with confirmed malaria in these hospitals since the launch of the Champions program, in comparison with the previous year.

## MATERIALS AND METHODS

### Study design.

This study conducted a secondary analysis of data from four rounds of OTSS in 12 of 16 hospitals participating in the Champions program. The remaining four facilities did not receive OTSS visits during the study period because of security concerns in their catchment area. One OTSS round was conducted before implementation of the Champions program (pre-Champions) and three rounds were conducted postimplementation (post-Champions round 1, post-Champions round 2, and post-Champions round 3). The timing for each round can be found in Table 1. The severe malaria checklist was completed by reviewing one or two inpatient records during an OTSS visit. The readiness checklist was completed once for each health facility during each OTSS visit. Specific data elements were extracted from the health facility readiness checklist (Supplemental Table 1) and the OTSS inpatient severe malaria checklist (Supplemental Table 2). In addition, data on malaria-related deaths and the number of malaria cases admitted in the 12 hospitals were extracted from the national HMIS data for 1 year before the implementation of the program (2020) and for 2 years post-Champions program implementation (2021 and 2022).

**Table 1:** Numbers of facilities and records reviewed from each round of OTSS

OTSS rounds	Number of facilities	Number of records reviewed	Records per facility
Pre-Champions (April–May 2021)	9	10	1–2
Post-Champions round 1 (August–November 2021)	11	14	1–2
Post-Champions round 2 (February 2022)	11	11	1
Post-Champions round 3 (September–October 2022)	11	11	1

OTSS = Outreach Training and Supportive Supervision.

### Measures and variables.

The OTSS facility readiness checklist was used to evaluate overall facility readiness and the availability of commodities, documentation, and materials to support the management of severe malaria cases. Facility readiness was defined as achieving an overall score of 90% or more on the health facility readiness checklist. In addition, subcomponents of the health facility readiness checklist, including availability of trained personnel, ACT availability for all age ranges, availability of injectable artesunate/artemether, availability of materials, and availability of documentation, were used in this analysis. The last two subcomponents were analyzed as continuous variables. The remaining subcomponents were analyzed as binary variables. Competency to manage severe malaria was defined as achieving a score of 90% or more on the inpatient severe malaria checklist. Key steps in the management of these cases were also assessed using composite indicators or subsections of the checklist. These included initial patient assessment and laboratory testing (patient assessment score) and prescribing of specific treatments for severe malaria (providing the right treatment) and its complications (hypoglycemia, severe anemia, and alteration of consciousness). These variables were left as continuous variables.

Malaria death and case fatality rates in the 12 hospitals were drawn from the national HMIS data validated by the government and were calculated for all sociodemographic groups combined. The malaria case fatality rate was defined as the number of malaria deaths divided by the total number of malaria cases admitted (by RDT and/or microscopy) to the 12 targeted hospitals.

### Analyses.

The team generated descriptive statistics for the overall facility readiness score (i.e., proportion achieving a score of ≥90%) and calculated the proportions for the single-item variables (availability of injectable artesunate/artemether and ACT per age group) and the average facility score for the composite variables (e.g., availability of documentation and materials).

The study team generated descriptive statistics for facility competency in the management of severe malaria (i.e., proportion achieving a score of ≥90%) and averages (sum of the scores divided by the number of records reviewed) for the different subcomponents in the management of severe malaria (patient assessment, providing the right treatment, and management of hypoglycemia, severe anemia, and alteration of consciousness).

Using the scores (dependent variables) for overall management of severe malaria, patient assessment, providing the right treatment, health facility readiness, material availability, and documentation availability, simple linear regressions were performed using Stata (v. 15.1) to assess the percent change between the OTSS pre-Champions round (baseline/reference group) and the post-Champions rounds 1, 2, and 3 (independent variables). Linear regressions were performed as opposed to a nonparametric Wilcoxon signed-rank test because only five facilities consistently received OTSS visits across all four rounds, greatly reducing the sample size for the Wilcoxon test. Regressions were deemed significant when the *P* value was less than 0.05.

Additionally, the study team performed the χ^2^ test to assess the relationship between overall deaths and the nonparametric Wilcoxon rank sum test to assess the difference between the case fatality rates from 2020 (reference), 2021, and 2022. These analyses were performed for 10 of the 12 targeted hospitals, as two of the hospitals were excluded because of missing data for more than 1 month during the period being assessed.

## RESULTS

Across the four rounds of OTSS, not all the facilities received an OTSS visit, which led to differing sample sizes ranging from 9 to 11 health facilities (Table 1). Overall, 46 record reviews were included in the analysis of the four rounds of OTSS data: 10 reviews for pre-Champions (April–May 2021), 14 reviews for post-Champions round 1 (August–November 2021), 11 reviews for post-Champions round 2 (February 2022), and 11 reviews for post-Champions round 3 (September–October 2022) (Table 1).

**Figure 1.**
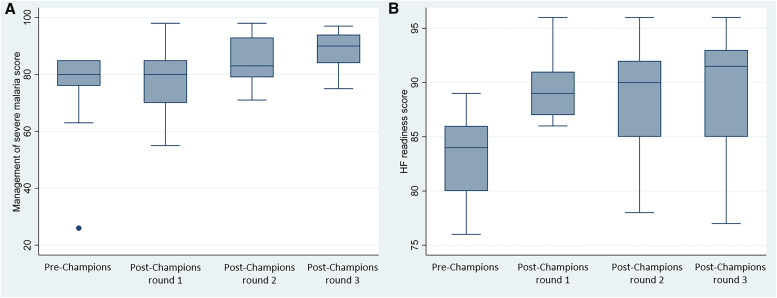
Box-and-whiskers plot of the management of severe malaria and health facility readiness across four rounds of Outreach Training and Supportive Supervision (OTSS). (**A**) Management of severe malaria. (**B**) Health facility readiness. Source: OTSS data, 2021–2022. OTSS = Outreach Training and Supportive Supervision.

Results for the key variables assessed are shown in Table 2 and Figure 1. The proportion of hospitals that reached a score of 90% or more in competency in the management of severe malaria and health facility readiness increased from 0% by pre-Champions to 50% and 50%, respectively, by post-Champions round 3. Average scores for patient assessment, providing the right treatment, availability of materials, and availability of documentation increased through all post-Champions rounds in comparison with those of the pre-Champions round.

**Table 2:** Scores for facility readiness, competency in the management of severe malaria, and components in pre-Champions and post-Champions OTSS rounds

Scores	Pre-Champions	Post-Champions round 1	Post-Champions round 2	Post-Champions round 3
Percentage of health facility observations scoring ≥90% in the management of severe malaria	0.0% *n* = 10	14.3% *n* = 14	36.4% *n* = 11	50.0% *n* = 10
Patient assessment (average)	56.7% *n* = 10	65.4% *n* = 14	72.9% *n* = 11	77.7% *n* = 10
Providing the right treatment (average)	85.7% *n* = 10	94.5% *n* = 14	95.4% *n* = 11	93.7% *n* = 10
Complications: hypoglycemia (average)	100.0% *n* = 1	100.0% *n* = 2	100.0% *n* = 2	NA
Complications: severe anemia (average)	96.0% *n* = 5	65.0% *n* = 4	92.0% *n* = 5	100.0% *n* = 6
Complications: alteration of consciousness (average)	0.0% *n* = 1	75.0% *n* = 4	100.0% *n* = 2	100.0% *n* = 4
Percentage of health facilities scoring ≥90% in the health facility readiness	0.0% *n* = 9	36.4% *n* = 11	54.5% *n* = 11	60.0% *n* = 10
Material availability (average)	84.1% *n* = 9	98.0% *n* = 11	100.0% *n* = 11	98.9% *n* = 9
Documentation availability (average)	83.2% *n* = 9	86.2% *n* = 11	90.7% *n* = 11	94.0% *n* = 10
ACT availability for infants (proportion)	88.9% *n* = 9	90.9% *n* = 11	100.0% *n* = 11	100.0% *n* = 10
ACT availability for children (proportion)	100.0% *n* = 9	100.0% *n* = 11	90.9% *n* = 11	100.0% *n* = 10
ACT availability for adolescents (proportion)	100.0% *n* = 9	81.8% *n* = 11	90.9% *n* = 11	90.0% *n* = 10
ACT availability for adults (proportion)	88.9% *n* = 9	81.8% *n* = 11	100.0% *n* = 11	90.0% *n* = 10
Injectable artesunate/injectable artemether availability (proportion)	100.0% *n* = 9	100.0% *n* = 11	90.9% *n* = 11	80.0% *n* = 10

ACT = artesunate- and artemisinin-based combination therapy; NA = no available data; OTSS = Outreach Training and Supportive Supervision.

A linear regression model demonstrated that in comparison with the pre-Champions scores, there were statistically significant increases in post-Champions scores for the overall management of severe malaria and health facility readiness as well as for average scores for subcomponents, such as patient assessment and material availability. For the management of severe malaria, regression analysis demonstrated modeled increases of +11% (*P* = 0.04) and +14% (*P* = 0.01), respectively, in post-Champions round 2 and post-Champions round 3 in comparison with pre-Champions (Table 3). There also were statistically significant increases in modeled patient assessment scores of +16% (*P* = 0.03) and +21% (*P* = 0.06) when post-Champions round 2 and post-Champions round 3, respectively, were compared to pre-Champions scores.

**Table 3:** Regression analysis of facility readiness and competency in the management of severe malaria comparing pre-Champions OTSS scores to post-Champions OTSS scores*

Variables	Pre-Champions	Post-Champions round 1 % change (95% CI)	Post-Champions round 2 % change (95% CI)	Post-Champions round 3 % change (95% CI)
Overall management of severe malaria (score)	Ref	3.3 (–7.0 to 13.6) *P* = 0.519	11.3 (0.4–22.1) *P* = 0.042	14.3 (3.2–25.4) *P* = 0.013
Patient assessment (score)	Ref	8.7 (–4.9 to 22.4) *P* = 0.204	16.2 (1.8–30.6) *P* = 0.029	21.0 (6.2–35.8) *P* = 0.006
Providing the right treatment (score)	Ref	8.8 (–0.6 to 18.2) *P* = 0.065	9.7 (–0.2 to 19.6) *P* = 0.056	8.0 (–2.1 to 18.1) *P* = 0.119
Health facility readiness score (score)	Ref	6.9 (2.5–11.3) *P* = 0.003	5.9 (1.5–10.3) *P* = 0.010	6.5 (2.0–11.0) *P* = 0.006
Material availability (score)	Ref	13.9 (7.6–20.2) *P* = 0.000	15.9 (9.6–22.2) *P* = 0.000	14.8 (8.3–21.3) *P* = 0.000
Documentation availability (score)	Ref	3.0 (–8.5 to 14.4) *P* = 0.604	7.5 (–4.0 to 19.0) *P* = 0.193	10.8 (–1.0 to 22.5) *P* = 0.071

CI = confidence interval; OTSS = Outreach Training and Supportive Supervision; Ref = reference variable: round 3.

* Regression analysis was not possible for ACT availability by age group as ACTs were available at almost all facilities across all rounds. Regression analysis also was not possible for complications (hypoglycemia, anemia, alteration of consciousness) due to small sample sizes.

The regression model for health facility readiness scores exhibited an increase for post-Champions rounds 1, 2, and 3 in comparison with pre-Champions of +7% (*P* = 0.003), +6% (*P* = 0.01), and +7% (*P* = 0.006), respectively (Table 3). The model for materials availability for all post-Champions rounds had a statistically significant increase compared with that for pre-Champions, of +14%, +16%, and +15%, respectively (*P* <0.001).

There were no statistically significant changes in the model pre-Champions to post-Champions in providing the recommended malaria treatment of severe malaria in national treatment guidelines, as the use of recommended treatments was high prior to the launch of the Champions program.

The associations between pre- and post-Champions program rounds of OTSS for additional variables such as complications (e.g., anemia, hypoglycemia, and conscience), ACT availability, and injectable artesunate/artemether availability could not be assessed because of insufficient sample size. Across all rounds, ACTs and injectable artesunate and/or artemether were available in nearly all facilities, preventing the regression analyses from being conducted.

### Trends in malaria deaths and case fatality rate in targeted hospitals.

For 10 of the participating hospitals, all malaria-related death reports as well as the total number of malaria cases admitted for all age groups were available from the HMIS and were used to calculate the case fatality rate for the January–December period of the years 2020, 2021, and 2022. For the period of the study, we observed that in 2022, the number of deaths was lower than for the years 2021 and 2020 (Table 4). The aggregate malaria case fatality rate across these 10 hospitals trended downward from 3.2% in 2020 to 3.1% in 2021 and 3.0% in 2023, although these reductions were not statistically significant (Table 4).

**Table 4:** Comparison of malaria death and case fatality rates for 10 participating hospitals in 2020 (pre-Champions program) to 2021 and 2022 (post-Champions program)*

Statistical test and statistic	2020	2021	2022
Chi-square test			
Number of deaths	510	514	332
Number of malaria cases admitted	16,140	16,337	11,114
χ^2^ (*P* value)	Ref	0.0049 (*P* = 0.944)	0.6551 (*P* = 0.418)
Wilcoxon signed-rank test			
Case fatality rate	3.2%	3.1%	3.0%
Median	3.2%	3.1%	3.0%
z-value (*P* value)	Ref	0.051 (0.9594)	1.070 (0.2845)

Ref = reference.

* Source: Cameroon National HMIS Data.

## DISCUSSION

This analysis of both OTSS and data in a limited sample of hospitals provides encouraging evidence of improvement in the quality of severe malaria management after implementation of the Champions program. Over the 2 years, as the program activities were implemented and reinforced, improvements in the overall management of cases and particularly patient assessment have increased and been sustained in comparison with the period before the launch of this program. Similarly, hospital readiness also improved, particularly the availability of materials, such as guidelines and job aids.

During the same period, hospital deaths decreased and the case fatality rate trended downwards, although these decreases were not statistically significant. The small numbers of recorded hospital deaths in the 10 hospitals where data were available limited the power of these analyses. The small sample also prevented controlling for other factors that might have impacted the malaria fatality rates, such as age and socioeconomic status of the patients, changes in transmission intensity, delays in care-seeking, changes in the availability of diagnosis and treatment of uncomplicated malaria, and the type of complications diagnosed. As this program is expanded, there will be additional opportunities to assess whether the improvements in the quality of case management for severe malaria can be linked to reductions in inpatient mortality and case fatality rate.

These results reinforce the findings of reports of similar interventions that have demonstrated the positive impact of quality improvement interventions on severe malaria case management.^14^^,^^15^ For example, in Nigeria, a 2021 project brief for the Support to the National Malaria Program Phase 2 (SuNMaP2) project reported on the improvement of inpatient malaria case management and health system readiness in 62 hospitals in six states where in-service programmatic interventions, like those implemented through the Champions program (such as in-service malaria case management trainings for health workers, distribution of national guidelines and job aids, and integrated supportive supervision), were implemented along with annual quality improvement cycles that include postassessment feedback, the creation of hospital quality improvement teams, and supportive follow-up visits.^15^ These activities produced a positive impact on the quality of care. From two assessment rounds undertaken in 2019 and 2020, injectable artesunate availability increased by +14% to +50% in four states, and its use for severe malaria increased in all states by +6% to +51%.^15^

Building on the lessons learned from other project experiences in quality improvement for severe malaria, the Champions program package deployed internal quality improvement interventions, including death audits and quarterly case reviews, reinforced by external quality improvement approaches such as mentoring and training and a robust monitoring approach. Other countries could learn from this approach to quality improvement of inpatient care for severe malaria and consider adopting it.

The primary limitation of this analysis was the small number of facilities and patient records and the limited time period assessed. Because the analysis included only 12 health facilities and a small number of records were reviewed, the study team was limited in the robustness of statistical analyses. Nonetheless, significant improvements in both readiness and competency were documented. Because of the small sample size, though, the effect of the program on the management of complications in these hospitals, which was the major focus of the training of the Champions, could not be assessed. As the Champions program is expanded to other facilities and additional experience is gathered in the existing facilities, future assessment of the program’s effect on management of complications will be warranted.

This analysis also relied on data collected through programmatic activities, which are potentially prone to data quality issues. However, the use of a digital platform to collect OTSS data likely mitigated some data quality issues. Using HMIS data to assess inpatient deaths and case fatality rates also has its limitations, including that malaria cases include all patients admitted who tested positive for malaria, which likely resulted in some patients with comorbidities being misclassified. Case-control studies may be able to better elucidate the link between these quality improvement activities and hospital mortality. Other limitations included nonrandom selection of districts and hospitals participating in the study and the lack of control facilities that were not implementing the Champions program. This analysis also could not control for other factors that might have affected malaria transmission and mortality, including changes in rain patterns, changes in availability of malaria diagnosis and treatment services, civil unrest, and other factors.

As management of the complications of severe malaria has often been neglected as a strategic priority, the Champions program has been an important additional intervention to supplement other quality improvement efforts, such as the OTSS, to address the quality of inpatient care for severe malaria and the high hospital mortality rate of malaria in Cameroon. The combination of training clinicians to become Champions, followed by internal quality improvement activities, and reinforcement by OTSS visits has the potential to make a significant difference in the quality of management of severe malaria cases and may reduce deaths from malaria. Further benefits to the quality of inpatient care of severe malaria and further reductions in inpatient deaths may be achieved if this Champions program is sustained and expanded to hospitals throughout Cameroon and in other countries.

## Supplemental Materials

10.4269/ajtmh.23-0528 Supplemental Materials
