# Supplementary material for: Impact of the Severe Malaria “Champions Program” on the Management of Severe Malaria Cases in 12 Hospitals of the North and Far North Regions of Cameroon
Source: Am J Trop Med Hyg. 2024 Feb 6;110(3 Suppl):76–82. doi: 10.4269/ajtmh.23-0528 (PMC10919230; doi:10.4269/ajtmh.23-0528)
Supplement: Supplemental Materials [file tpmd230528.SD1.pdf]

SUPPLEMENTAL MATERIALS

S1. Components of facility readiness checklist.

**Information**	**Score**
Does facility offer ANC services?	NS
Does facility distribute LLINs to children under 5 years of age?	NS
Does facility offer laboratory services?	NS
Does facility offer diazepam?	NS
Does facility offer blood transfusion?	NS
Is there at least 1 CHW attached to this facility?	NS
Does facility offer case management of severe malaria services?	NS
**Availability of medications**<br>Are these medications available today?	
Paracetamol	1
Sulfadoxine-Pyrimethamine	2
ACTs for children under 5 years	2
ACTs for children 5-7 years	2
ACTs for children 7-13 years	2
ACTs for children over 13 and adults	2
Injectable Quinine	2
Compressed Quinine	2
Injectable Artesunate and/or IM Injectable Artemether	2
Artesunate Rectocaps	2
Iron Tablets	1
Diazepam	1
5% Glucose	2
10% Glucose	1
30% Glucose	1
Lactate Ringer	1
Saline Solution	1
**Availability of commodities**<br>Are these commodities available today?	
Oxygen	3
Drinking water at the site of ANC	2
Disposable glasses available (or consistent cleaning) for the DOT strategy of the SP during ANC	1
Examination gloves	2
Soap or hand sanitizer gel	1
Sharps containers	3
Thermometer	2
Personal scale	2
Syringes	2
Alcohol	1

Cotton	1
Malaria RDTs	3
LLINs	3
Reagents for determining blood groups	2
Blood for transfusion	1
**Availability of documentation**<br>Are these documents available today?	
Records of curative consultations	2
Records of prenatal consultations	2
Records of the distribution of LLINs to children under 5 years of age	1
Records of the distribution of LLINs to pregnant women	1
HMIS reports	2
Monthly CHW reports	2
Manual for filling out the data collection media	1
**Availability of materials**<br>Are these materials (guides, job aides, etc) available today?	
Malaria and pregnancy guide or tools/support on ANC	1
ANC guidelines	1
National guidelines for the management of malaria	2
Management of uncomplicated malaria fact sheet	1
Management of severe malaria fact sheet	1
Management of malaria in pregnancy fact sheet	1
Preparation of artesunate injection PEC fact sheet	1
LLIN distribution guidelines	1
**Availability of trained human resources**	
Does the health facility have at least 50% of staff trained to manage malaria cases using the latest national guidelines?	3
**Availability of quality data**	
**Number of pregnant women received in ANC 1**	
Amount reported (R)	NS
Amount found in the register (F)	NS
Difference (F-R) <= 10%	1
**Number of pregnant women who received IPT 3**	
Amount reported (R)	NS
Amount found in the register (F)	NS
Difference (F-R) <= 10%	1
**Number of suspected malaria cases registered in the health facility**	
Amount reported (R)	NS
Amount found in the register (F)	NS
Difference (F-R) <= 10%	1
**Number of suspected malaria cases tested by RDT or thick smear test**	
Amount reported (R)	NS
Amount found in the register (F)	NS

Difference (F-R) <= 10%	1
**Number of uncomplicated malaria cases tested by RDT or thick smear test**	
Amount reported (R)	NS
Amount found in the register (F)	NS
Difference (F-R) <= 10%	1
**Number of confirmed uncomplicated malaria cases treated with ACTs**	
Amount reported (R)	NS
Amount found in the register (F)	NS
Difference (F-R) <= 10%	1
**Number of ACT treatments consumed (taken from the health facility's pharmacy)**	
Amount reported (R)	NS
Amount found in the register (F)	NS
Difference (F-R) <= 10%	1

S2. Components of inpatient severe malaria checklist.

Collecting historical patient information	Score
Did the provider record the patient's age?	1
Is the patient over 12 years old?	NS
Is the patient a woman of childbearing age?	NS
Did the provider record where the patient lived?	1
Did the provider record whether the patient was referred from a peripheral facility or not?	1
Was the patient referred?	NS
If the patient was referred, has the patient received pre-transfer treatment in accordance with national guidelines noted in the register?	1
If the patient is a woman of childbearing age, has her pregnancy status been verified?	2
If the patient is pregnant, what is the determined gestational age?	NS
**Patient Assessment**	
Did the provider take the patient's temperature?	2
Did the provider measure the patient's weight?	2
Did the provider take the patient's blood pressure?	2
Did the provider take the patient's pulse?	2
Did the provider take the patient's respiratory rate?	2
Were signs of severe malaria found in the patient (behavioral changes, convulsions, decreased urine output or dark urine, spontaneous bleeding, prostration/generalized weakness, inability to drink, respiratory distress, jaundice, altered consciousness) noted in the register?	2
Did the provider record the information collected during the complete physical examination of the patient (eyes, conjunctivae, fundus, palms, ears, throat, neck, heart, lungs, abdomen, and reflex)?	1
Has the provider confirmed the diagnosis of severe malaria based on the positivity of the GE/FS blood smear or RDT biological test?	2
Was microscopy performed?	NS
If microscopy was performed, was the parasite density specified?	1
Has the provider ordered a Complete Blood Count (CBC) or hemoglobin (Hb) count to confirm severe anemia in the patient?	3
Did the provider request a Blood Glucose test to confirm hypoglycemia in the patient?	3
Did the provider request Uremia and Creatinine tests to confirm renal failure in the patient?	2
Has the provider performed a urine dipstick test for hemoglobinuria?	2
Did the provider request a blood gas test to confirm metabolic acidosis in the patient?	1
Did the provider note the results of the biological tests requested in the registry?	1
**Providing the right treatment**	
Did the provider use artesunate injection to treat severe malaria?	3
If Yes, has the provider correctly calculated the amount of artesunate required based on the patient's weight?	3
Did the provider follow the procedures for the preparation of the artesunate injection?	3
Check that the provider follows the procedure for the preparation of the artesunate	NS

injection	
i- Removed and injected the entire contents (1 ml) of the sodium bicarbonate ampoule into the artesunate powder vial	NS
ii- shook gently until dissolved and a clear reconstituted solution was obtained	NS
iii- removed and injected the required volume of 5% saline or dextrose solution (depending on IV or IM) into the reconstituted artesunate solution	NS
Did the provider administer a weight-appropriate dose of artesunate based on IV or IM route?	3
Has the provider properly defined the time interval between doses to be injected into the patient for each day?	2
Did the provider give the patient an antipyretic to treat the fever?	1
Did provider use an artemether injection to treat severe malaria?	NS
If the provider has used an artemether injection to treat severe malaria, is the dosage according to the guidelines?	3
Did provider use a quinine injection to treat severe malaria?	NS
If the provider has used a quinine injection to treat severe malaria, is the dosage correct?	3
Did the provider adhere to the time limit of four hours or more to infuse the quinine salt?	2
Did the patient receive antimalarial relay treatment after the emergency passed?	2
If yes, does the relay treatment used comply with national guidelines?	2
Does the provider carry out regular parasitological monitoring through testing the GE and parasite density?	1
**Management of Altered Consciousness**	
Is the patient experiencing altered consciousness/ coma?	NS
Has the patient received appropriate nursing care (aspiration, clearing of airways, mobilization in bed and lying down if necessary, etc.)?	3
Is the patient's progress assessed regularly (vital signs, level of consciousness, Glasgow scale, glucose, Hb)?	3
Did patient have a seizure?	NS
If the patient had a seizure, did the provider administer diazepam?	3
**Management of Severe Anemia**	
Does the patient have severe anemia?	NS
Is the decision to prescribe or provide a blood transfusion appropriate?	3
Has the provider calculated the amount of blood to be administered according to standards?	3
Has the provider determined the patient's blood type and rhesus?	3
Has the provider completed the pre-transfusion compatibility test?	3
Does the provider plan to check the hemoglobin level regularly?	3
**Management of Hypoglycemia**	
Does the patient have hypoglycemia?	NS
Did the provider administer IV glucose (G10, G30) according to national guidelines?	3
Does the provider monitor the patient's blood sugar levels regularly?	3
**Management of Metabolic Acidosis**	
Does the patient have metabolic acidosis?	NS
Is blood gas prescribed or performed?	3

Is oximetry used to monitor oxygen saturation?	3
Have appropriate measures been taken to correct acidosis (electrolyte balance, rehydration, etc.)?	3
**Dehydration/ Hypovolemia**	
Does the patient have dehydration?	NS
Did the patient receive sufficient IV fluids according to standards?	3
Was the admission/discharge ratio monitored regularly until the patient stabilized?	3
**Other Complications**	
Does the patient have a complication other than those listed above?	NS
Have appropriate measures been taken to correct other complications found in the patient (acute renal failure, pulmonary edema, etc.)?	3

NS= Not scored
